# Mutation of the BRCA1 SQ-cluster results in aberrant mitosis, reduced homologous recombination, and a compensatory increase in non-homologous end joining

**DOI:** 10.18632/oncotarget.4876

**Published:** 2015-08-12

**Authors:** Jason M. Beckta, Seth M. Dever, Nisha Gnawali, Ashraf Khalil, Amrita Sule, Sarah E. Golding, Elizabeth Rosenberg, Aarthi Narayanan, Kylene Kehn-Hall, Bo Xu, Lawrence F. Povirk, Kristoffer Valerie

**Affiliations:** ^1^ Department of Radiation Oncology, Virginia Commonwealth University, Richmond, VA 23298, USA; ^2^ Department of Biochemistry and Molecular Biology, Virginia Commonwealth University, Richmond, VA 23298, USA; ^3^ National Center for Biodefense and Infectious Diseases, George Mason University, Manassas, VA 20110, USA; ^4^ Cancer Research Department, Southern Research Institute, Birmingham, AL 35205, USA; ^5^ Department of Pharmacology and Toxicology, Virginia Commonwealth University, Richmond, VA 23298, USA; ^6^ Massey Cancer Center, Virginia Commonwealth University, Richmond, VA 23298, USA

**Keywords:** radiation, DNA damage, DNA repair, phosphorylation, cell cycle

## Abstract

Mutations in the breast cancer susceptibility 1 (*BRCA1*) gene are catalysts for breast and ovarian cancers. Most mutations are associated with the BRCA1 N- and C-terminal domains linked to DNA double-strand break (DSB) repair. However, little is known about the role of the intervening serine-glutamine (SQ) - cluster in the DNA damage response beyond its importance in regulating cell cycle checkpoints. We show that serine-to-alanine alterations at critical residues within the SQ-cluster known to be phosphorylated by ATM and ATR result in reduced homologous recombination repair (HRR) and aberrant mitosis. While a S1387A BRCA1 mutant - previously shown to abrogate S-phase arrest in response to radiation - resulted in only a modest decrease in HRR, S1387A together with an additional alteration, S1423A (BRCA1^2P^), reduced HRR to vector control levels and similar to a quadruple mutant also including S1457A and S1524A (BRCA1^4P^). These effects appeared to be independent of PALB2. Furthermore, we found that BRCA1^4P^ promoted a prolonged and struggling HRR late in the cell cycle and shifted DSB repair from HRR to non-homologous end joining which, in the face of irreparable chromosomal damage, resulted in mitotic catastrophe. Altogether, SQ-cluster phosphorylation is critical for allowing adequate time for completing normal HRR prior to mitosis and preventing cells from entering G1 prematurely resulting in gross chromosomal aberrations.

## INTRODUCTION

Cells harboring specific mutations in *BRCA1* are defective in DNA double-strand break (DSB) repair, cell cycle checkpoints, and transcriptional regulation which lead to genomic instability and malignancy [1]. BRCA1 was initially believed to facilitate and enforce different types of DNA repair through a single large BRCA1-associated protein complex, however, many studies conducted over the last decade suggest that BRCA1 coordinates a very sophisticated multi-factorial DNA damage response (DDR). It is now clear that BRCA1 plays different and unique roles in the spatiotemporal interactions with critical DDR proteins via specific BRCA1 domains that regulate cell cycle checkpoints and DSB repair [2, 3].

Both homologous recombination repair (HRR) and non-homologous end joining (NHEJ) are regulated by BRCA1 in a coordinated process through protein-protein interactions associated mostly with the N- and C-terminal domains. The ubiquitin ligase BARD1 interacts with the N-terminal BRCA1 domain and ubiquitinates specific proteins that bind to the BRCT domain located at the C-terminus of BRCA1 [4, 5]. At least three distinct BRCA1 protein complexes - referred to as complexes A, B, and C - are able to bind the BRCT domain through phospho-serine-X-X-phenylalanine (pS-X-X-F) motifs (where “X” represents any amino acid), and have been reported as being important in both checkpoint activation as well as DSB repair ([2] and references therein). The “A complex”, which consists of Abraxas forming an intermediate binding partner between BRCA1 and RAP80, is important for directing BRCA1 to DSBs through histone ubiquitination and phosphorylation chromatin modifications, as well as the regulation of DNA resection [6–11]. During HRR, RAD51 binds to single-stranded DNA (generated at DSBs via DNA end resection), a step crucial for DNA strand invasion and making contact with a sister chromatid [12]. “Complex B”, containing BACH1, interacts with RAD51 and is important for keeping RAD51 loaded onto the DNA strand during S-phase, a phase of the cell cycle where this complex is particularly important [13, 14]. Finally, the “C complex” is formed with one of the better-understood BRCT-binding partners, CtIP. At sites of DSBs, the BRCA1-CtIP-MRN complex is pivotal for 3′- to - 5′ DNA end resection, thus facilitating HRR [15, 16]. Additionally, BRCA1 interacts with the protein PALB2 through its centrally-located Coiled-coil domain [17–19]. PALB2 recruits BRCA1 to sites of DNA damage and serves as an anchoring binding partner with BRCA2, thus facilitating the conglomeration of BRCA1, BRCA2, and RAD51 to form Holliday junctions. This recruitment provides a plausible mechanism by which BRCA1 and BRCA2 interact at the DSB in their capacity as important regulators of HRR.

Near the tandem BRCT repeats and close to the Coiled-coil domain is the SQ-cluster domain, a serine- and threonine-rich region which is phosphorylated by the ataxia telangiectasia mutated (ATM) and ataxia telangiectasia and RAD3-related (ATR) kinases [20–22]. Serine residues 1423 and 1524 are redundantly phosphorylated by ATM and ATR, whereas S1387 is a specific target for ATM and S1457 for ATR [20, 23]. These modifications have direct consequences for cell cycle control. We have previously demonstrated that kinase activity directed towards S1387 has been implicated in activating the intra-S checkpoint while phosphorylation of S1423 and S1524 is important for enacting G2/M arrest in response to radiation [24, 25]. After the SQ-domain has been phosphorylated by ATM/ATR and the S/G2/M checkpoints have been activated, BRCA1 goes on to coordinate the repair process by modulating DNA resection and facilitating which type of DSB repair (HRR vs. NHEJ) will occur.

Serine modifications outside of the SQ-domain are also important in regulating the DDR. Specifically, the CHK2 kinase phosphorylates S988 in response to microtubule damage [26]. This post-translational modification triggers mitotic arrest via inhibition of the microtubule nucleation activity of BRCA1, thus preventing the proper transition to (and through) mitosis. Interestingly, phosphorylation of S988 has also been implicated in DSB repair [27]. Although disparate studies have suggested complementary roles for BRCA1 modifications in regulating checkpoint activity and the DDR, it is currently unknown if ATM- and ATR-mediated phosphorylation of the BRCA1 SQ-cluster influences mitosis and/or the quality of DSB repair. In the present study we show that SQ-cluster serine-to-alanine alterations result in a shift from HRR to NHEJ and the abrogation of the G2/M checkpoint. As a result, this causes inappropriate entry into mitosis and the formation of mitotic aberrations, leading to subsequent chromosomal abnormalities and cell death via mitotic catastrophe.

## RESULTS

### Progressive BRCA1 SQ-cluster serine-to-alanine alterations result in reduced HRR

**Figure 1:**
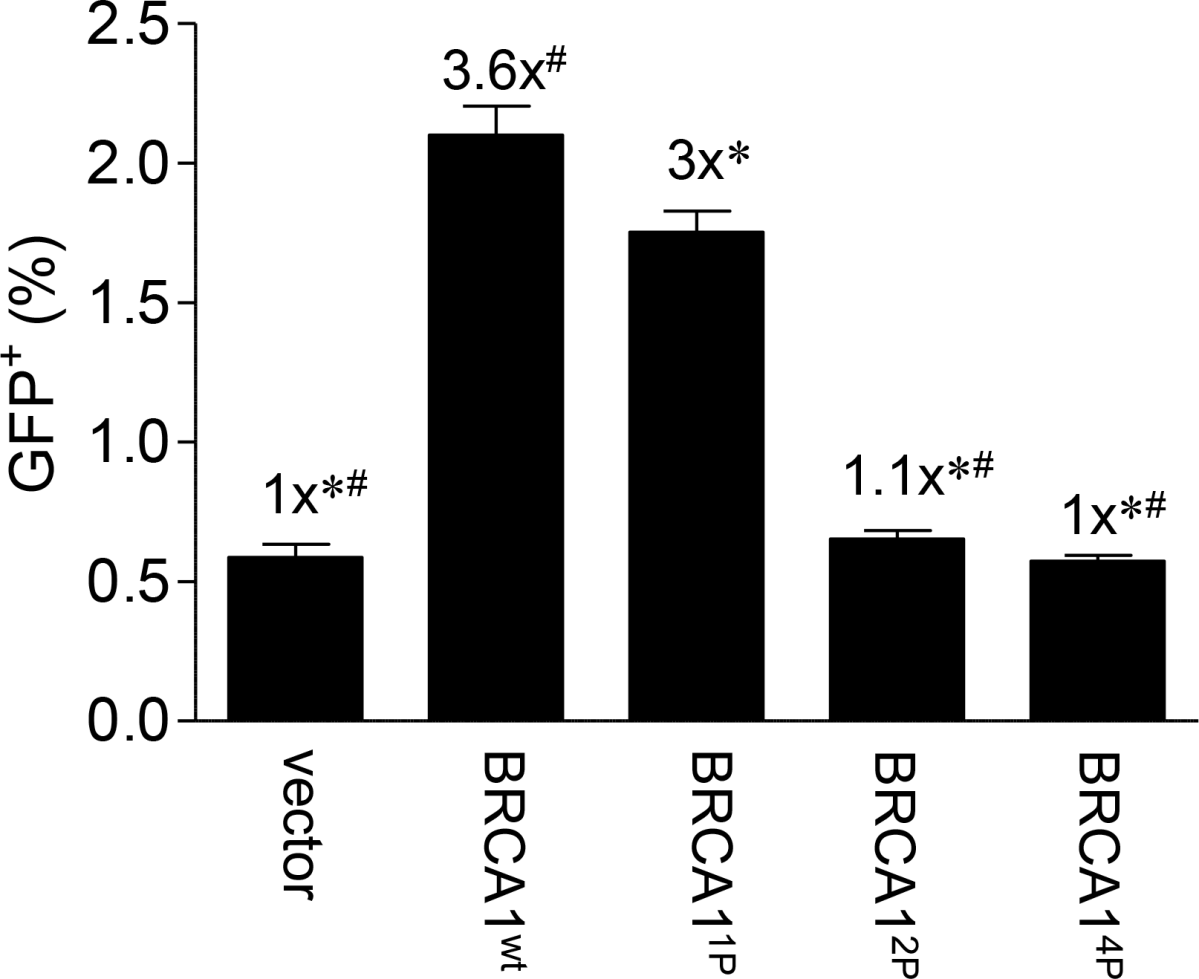
Progressive mutagenesis of the BRCA1 SQ-cluster results in reduced HRR HCC1937/DR-GFP cells were infected with Ad-SceI 48 hours post-infection with the indicated HD-Ad vectors. Thirty-thousand cells were assayed for GFP fluorescent events by flow cytometry 72 hours after Ad-SceI infection. Confirmation of quantitative infection was done using β-galactosidase staining co-expressed from the virus (data not shown) [28] and western blotting which demonstrated equal expression of the exogenous BRCA1 wild-type and mutant proteins (Supplemental Figure 1). Error bars show the SEM from 3 independent experiments. *F*(4,10) = 134.8, *p* = < 0.0001. *p < 0.05 relative to BRCA1^wt^, ^#^*p* < 0.05 relative to BRCA1^1P^.

To determine the importance of BRCA1 phosphorylation in HRR, we constructed adenoviruses expressing mutant BRCA1 with key serine residues changed to alanine and infected these into HCC1937 cells harboring the DR-GFP repair reporter as reported previously [28]. The HCC1937 cells, expressing a truncated BRCA1, have been used extensively in the past to determine the role of BRCA1 in HRR [29, 30]. These experimental conditions create transient, physiological levels of BRCA1 in a majority of treated cells which appear to behave in a manner more conducive to normal cell function, as compared to cells experiencing over-expression of BRCA1 from plasmids in transient transfections [28]. S1387 was chosen as a starting point because our previous work revealed an ATM-dependent S-phase checkpoint when this site was mutated [23, 25]. It is well established that HRR is very important during late S-phase. In addition to the S1387A single mutant (denoted 1P), we constructed a S1387/1423A double mutant (BRCA1^2P^) as well as a S1387/1423/1457/1524A quadruple mutant (BRCA1^4P^) in which ATM- and ATR-targeted serine residues are mutated to alanines. Whereas the S1387A mutant resulted in a modest but significant decrease in HRR (~17%) relative to BRCA1 wild-type, the additional alterations involving S1423 in the BRCA1^2P^ and BRCA1^4P^ constructs resulted in significantly reduced levels of HRR, similar to what was observed in vector control cells (Figure 1).

A previous study demonstrated that single S1387A, S1423A, and S1524A BRCA1 mutants still bound PALB2 necessary for HRR [18]. In order to determine whether the reduced HRR seen in our system was caused by the disruption of the PALB2-BRCA1 interaction, we performed co-immunoprecipitations with extracts from HEK293T cells co-transfected with plasmids expressing the various phospho-mutants as well as PALB2. We found that the binding of PALB2 to BRCA1 was unaffected by mutating one, two, or all four critical serine residues to alanines in the SQ-cluster (Supplemental Figure 2), consistent with reports by others separating PALB2 binding to the Coiled-coil domain from single SQ-cluster alterations [17, 18]. Altogether, these findings suggest that phosphorylation of the BRCA1 SQ-cluster is critical for HRR but independent from its interaction with PALB2.

### Quadruple BRCA1 (4P) mutant sensitizes ovarian UWB1.289 cells to mitomycin C

**Figure 2:**
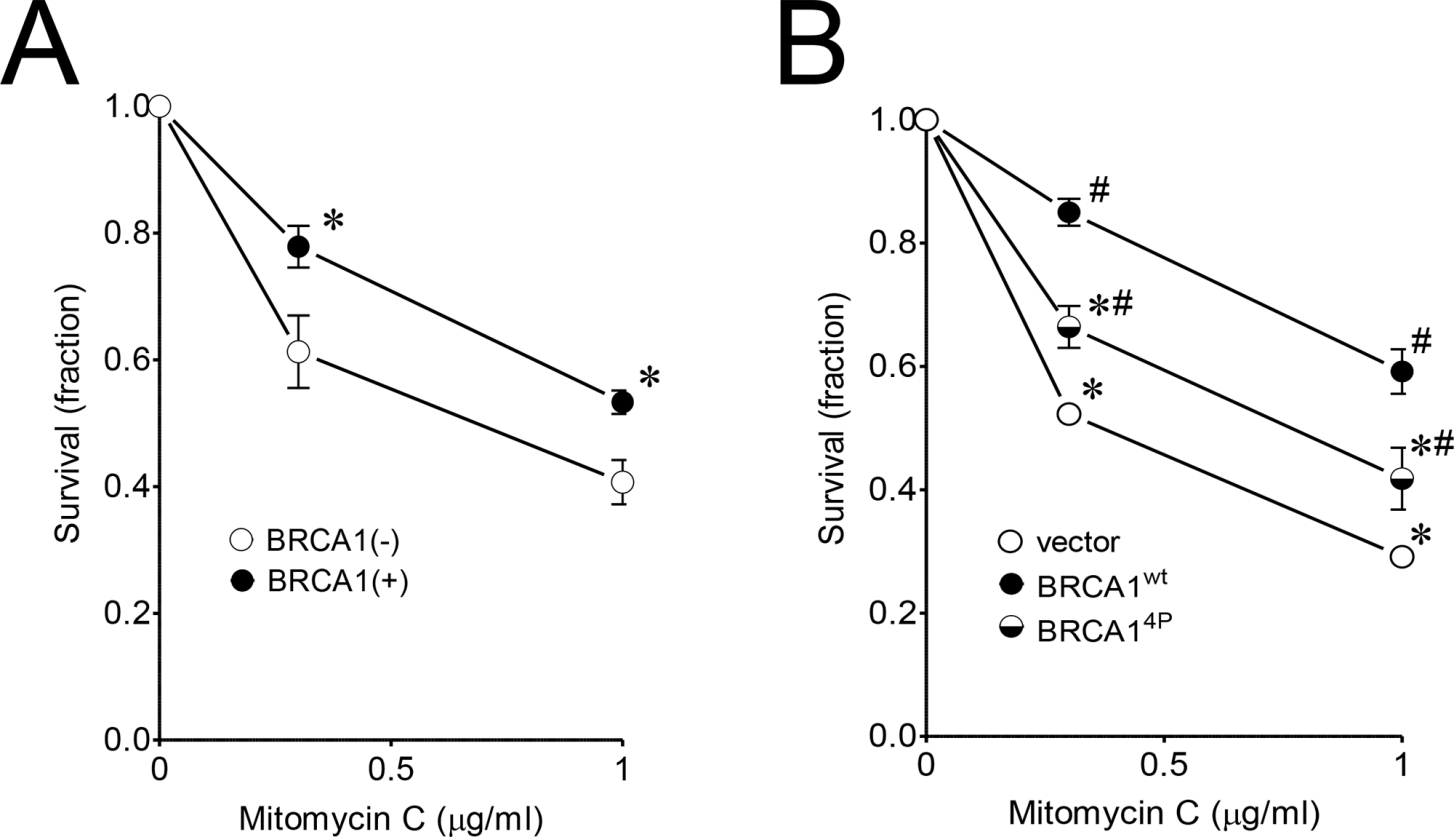
Quadruple BRCA1 (4P) mutant sensitizes ovarian UWB1.289 cells to mitomycin C **A.** UWB1.289 (+/−) BRCA1 complemented cells were treated with 0.3 and 1 μg/ml mitomycin C for 3 days. Untreated control cells were set to *a* value of 1. Error bars show the SEM from 4 independent experiments. *p* = 0.0458 and 0.0186 for 0.3 and 1 μg/ml mitomycin C treatment, respectively, when BRCA1 complemented (+) and uncomplemented (−) cells were compared. *p < 0.05 relative to BRCA1(−) cells at each dose. **B.** UWB1.289 cells were infected with the indicated HD-Ad vectors. Twenty-four hours after infection, cells were treated with 0.3 and 1 μg/ml mitomycin C for 4 days. Error bars show the SEM from 4 independent experiments. *F*(2,8) = 41.01, *p* = < 0.0001 and *F*(2,9) = 17.56, *p* = 0.0008 for 0.3 and 1 μg/ml mitomycin C treatment, respectively. *p < 0.05 relative to BRCA1^wt^ and ^#^*p* < 0.05 relative to vector control at each dose.

Having found that BRCA1^4P^ with all of the ATM/ATR targets mutated resulted in a severe HRR defect, we decided to use this mutant for examining the global impact from loss of function of the SQ-cluster in our subsequent experiments, beginning with the effect on cell survival following mitomycin C treatment. Mitomycin C causes inter-strand cross-link (ICL) damage which requires HRR [31]. Human ovarian UWB1.289 cells were recently reported to express a truncated BRCA1 (stop codon at codon 845) compared to the smaller truncation in HCC1937 (erroneous translation distal to codon 1775) and therefore serve as an alternative and more suitable cell model for examining BRCA1 function since the entire SQ-cluster is missing in UWB1.289 cells [32]. A matched BRCA1-complemented cell clone with a near-normal BRCA1 phenotype was also described. Thus, we exposed the UWB1.289 (−/+) BRCA1 cell pair to mitomycin C and found that the BRCA1-complemented cells were able to significantly reduce drug toxicity and increase survival (Figure 2A). We then infected the UWB1.289 parental cells with either wild-type or BRCA1^4P^ virus and found that BRCA1^4P^ only partially rescued survival (Figure 2B), even though virus infection was equal and the wild-type and mutant alleles were expressed at similar levels (Supplemental Figure 3A and 3B). These results support the repair reporter data in showing that BRCA1^4P^ severely impairs HRR.

### Inability to phosphorylate BRCA1 at SQ-sites results in prolonged and struggling HRR late in the cell cycle

**Figure 3:**
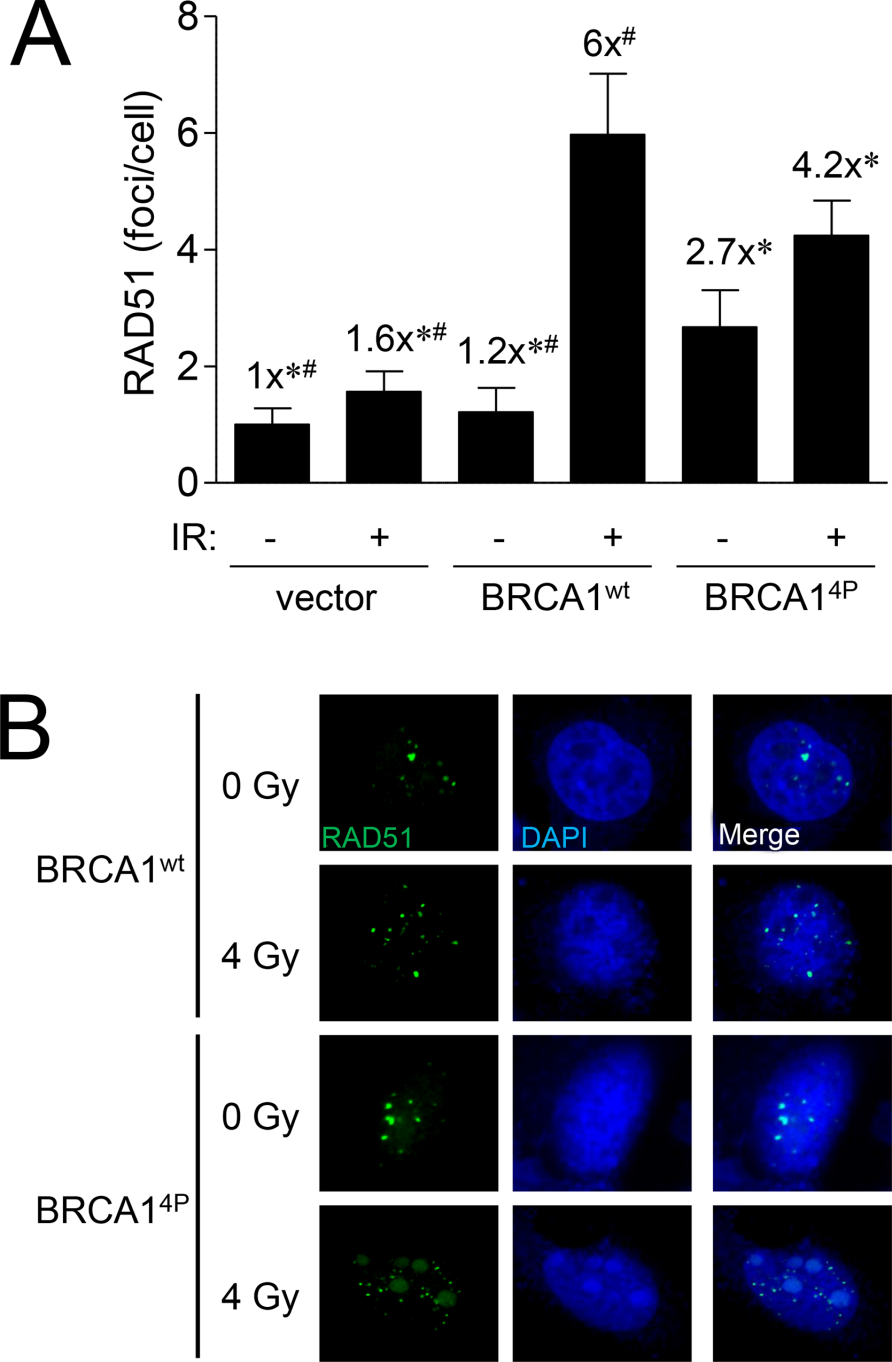
BRCA1 (4P) increases the basal level of RAD51 foci and reduces radiation-induced foci **A.** UWB1.289 cells were infected with the indicated HD-Ad vectors. Forty-eight hours after infection, cells were exposed to 4 Gy or left un-irradiated and fixed 8 hours after IR treatment. RAD51 foci in at least 25 cells were counted for each group. Error bars show the SEM. *F*(5,205) = 11.32, *p* = < 0.0001. *p < 0.05 relative to BRCA1^wt^ (+) IR, ^#^*p* < 0.05 relative to BRCA1^4P^ (+) IR. **B.** Representative images of cells used to generate the data presented in panel A immunolabeled with RAD51 antibody (*green*) and counter-stained with DAPI (*blue*) to label cell nuclei. Images were acquired at 63x power.

To further characterize the HRR defect seen with BRCA1^4P^, we next examined RAD51 foci formation in UWB1.289 cells following ionizing radiation (IR) treatment as a measure of HRR competency. Our previous work showed that certain BRCA1 BRCT mutants demonstrated aberrant hyper-recombination indicated by the surprisingly high RAD51 and RPA foci levels seen in unirradiated cells [28]. Whereas that study focused on distinctly different *BRCA1* mutations, our results at this point in the current study led us to believe that BRCA1^4P^ might behave differently. Indeed, cells expressing wild-type BRCA1 resulted in significantly more RAD51 foci formation following IR as expected whereas BRCA1^4P^ only partially rescued the repair defect (Figure 3A). However, we also noticed a trend of increased RAD51 foci in untreated cells expressing BRCA1^4P^, suggesting that these cells have an uncoordinated repair response and accumulate more spontaneous DNA damage relative to wild-type BRCA1 cells. In addition, large nuclear structures lightly stained with DAPI harboring RAD51 were observed in irradiated cells expressing BRCA1^4P^ - but not wild-type BRCA1 - which may be further indication of impaired HRR (Figure 3B). However, these structures were not promyelocytic leukemia bodies (data not shown) as were found in our previous study with BRCT mutant cells [28]. Altogether, BRCA1^4P^ cells display aberrant RAD51 foci and dysfunctional HRR.

**Figure 4:**
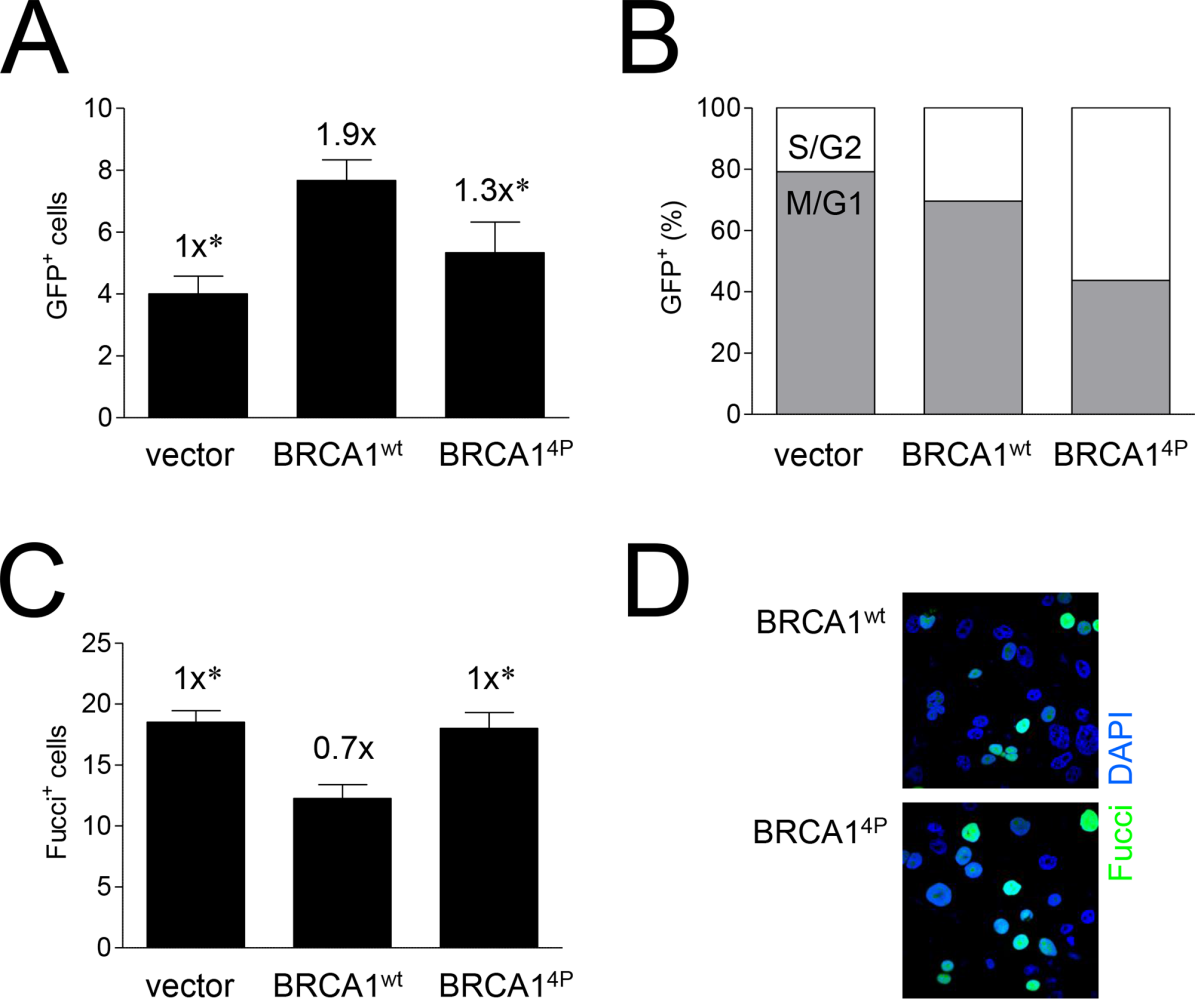
Mutations of BRCA1 phosphorylation sites increase the time cells undergoing HRR remain in the later stages of the cell cycle **A.** HCC1937/DR-GFP cells were infected with the indicated HD-Ad vectors followed by infection with Ad-SceI 48 hours later, and cell fixing 48 hours after Ad-SceI infection. GFP^+^ (HRR) cells in 6 (six) 40x fields were counted for each group. Error bars show the SEM. *F*(2,15) = 5.886, *p* = 0.0130. *p < 0.05 relative to BRCA1^wt^. **B.** Percentage of GFP^+^ cells from panel A co-expressing cyclin A. Cyclin A is expressed in S and G2 but not M phase; GFP^+^/cyclin A^−^ cells are thus in M or G1. Representative images are shown in Supplemental Figure 4. **C.** UWB1.289/Fucci cells were infected with the indicated HD-Ad vectors, fixed 48 hours post-infection, and Fucci^+^ cells (at least 9 per 40x field with 4–8 fields imaged for each group) were counted. Error bars show the SEM. *F*(2,17) = 8.124, *p* = 0.0033. *p < 0.05 relative to BRCA1^wt^. **D.** Representative images of cells used to generate the data presented in panel C showing Fucci^+^ (*green*) fluorescent cells and DAPI (*blue*) staining to label cell nuclei. Representative images of Fucci^+^/cyclin A^−^ cells in M phase are shown in Supplemental Figure 5.

To investigate the underlying mechanism for the HRR defect in BRCA1^4P^ cells, we then examined whether there were cell cycle differences relative to wild-type BRCA1. We, and others, have demonstrated that cells expressing BRCA1 SQ-cluster phospho-serine mutants possess defective cell cycle checkpoints [20, 24, 25]. Examination of individual HCC1937/DR-GFP cells undergoing HRR by fluorescence microscopy showed that BRCA1^4P^ cells had overall fewer GFP^+^ cells (Figure 4A), but the fraction of GFP^+^ cells that were cyclin A^+^ (S and G2 cells) was higher for BRCA1^4P^ than wild-type BRCA1 cells (Figure 4B, Supplemental Figure 4). This result suggests that although HRR occurs at low levels in cells expressing BRCA1^4P^, these cells accumulate or stall at the later stages of the cell cycle. Differences in the cell cycle distribution of individual cells were further examined using the Fucci reporter which marks cells residing outside of the G1 phase including M [33]. Thus, cyclin A^+^ cells are always Fucci^+^ whereas a fraction of Fucci^+^ cells are not cyclin A^+^, indicating that these cells have entered mitosis (Supplemental Figure 5). We found that after infection with the BRCA1^4P^ virus, ~30% more cells were Fucci^+^ and therefore remained longer in S/G2/M compared to wild-type BRCA1 cells (Figure 4C and D). These results demonstrate that BRCA1^4P^ cells not only have an impaired G2/M checkpoint but also abnormal and prolonged mitosis, even in the absence of DNA damage.

### Cells unable to phosphorylate BRCA1 SQ-sites have prolonged mitosis and display increased mitotic aberrations

**Figure 5:**
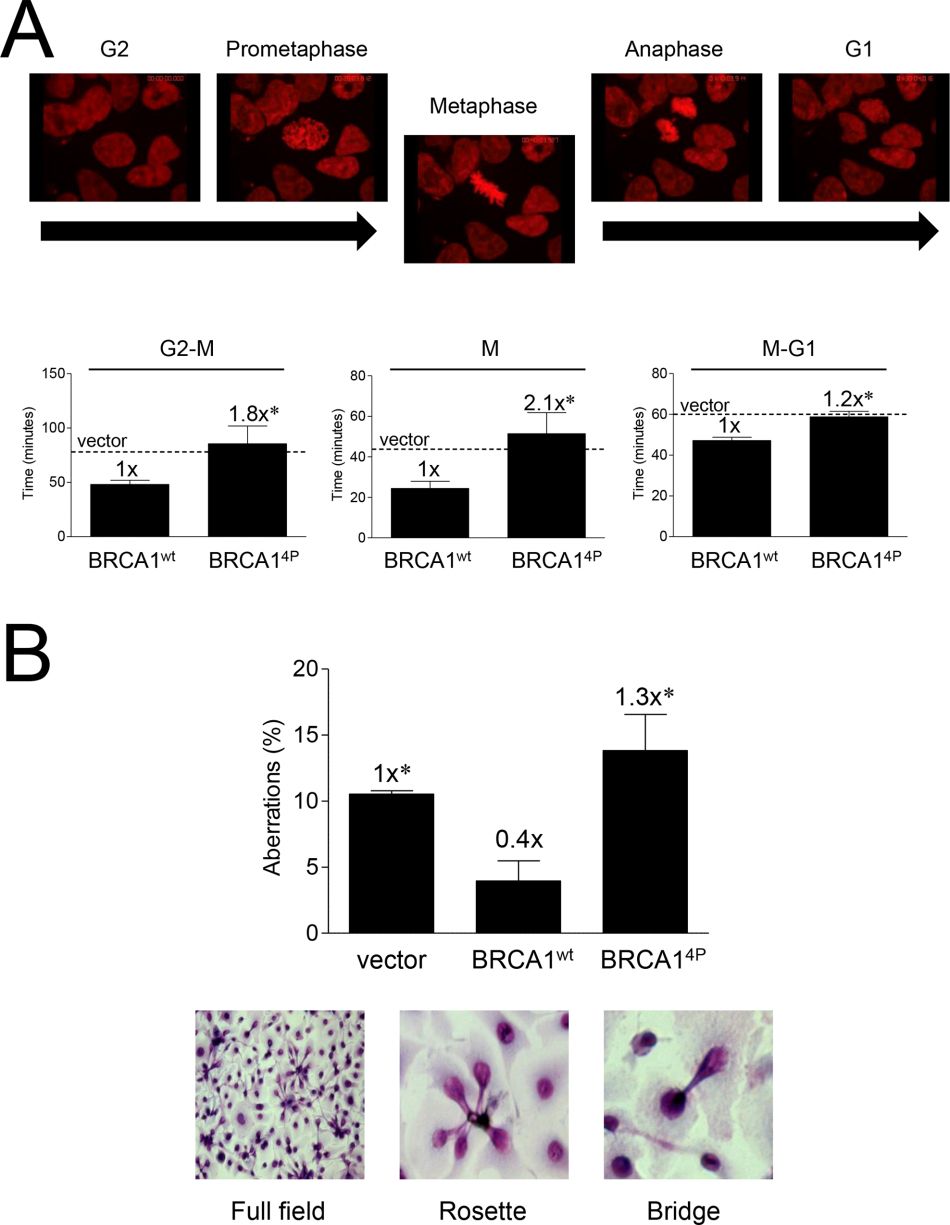
Mutations of BRCA1 phosphorylation sites prolong mitosis and induce mitotic aberrations **A.** UWB1.289/H2B-mCherry (*red*) fluorescent cells were infected with the indicated HD-Ad vectors and subjected to live-cell video imaging throughout the indicated mitotic phases 48 hours post-infection. Representative still images from BRCA1^wt^ cells are shown (for complete recording examples of cells undergoing normal and prolonged, aberrant mitosis, see Supplemental Figure 6). Dashed lines indicate average vector control levels. Error bars show the SEM. *p* = 0.0454 for G2 to metaphase, *p* = 0.0348 for metaphase, and *p* = 0.0015 for metaphase to G1. *p < 0.05 relative to BRCA1^wt^. **B.** UWB1.289 cells were infected with the indicated HD-Ad vectors and cells were fixed and Giemsa stained 72 hours post-infection. Aberrations were scored as the total number of rosette bundles and bridges relative to normal nuclei. Representative *brightfield* images are shown. At least 200 cells were counted for each group. Error bars show the SEM from 3 independent experiments. *F*(2,6) = 7.647, *p* = 0.0224. *p < 0.05 relative to BRCA1^wt^.

To further investigate cell cycle differences, we examined histone H2B-mCherry-expressing UWB1.289 cells infected with BRCA1^4P^ or wild-type virus as they progressed through mitosis using live-cell confocal imaging. In the absence of any DNA damage, we found that BRCA1^4P^ cells spent significantly more time in all phases of mitosis relative to BRCA1 wild-type (Figure 5A). This effect was most pronounced in the progression towards metaphase, as well as within metaphase itself (video recordings of cells undergoing normal and prolonged, aberrant mitosis, see Supplemental Figure 6). Furthermore, we found that while cells complemented with wild-type BRCA1 demonstrated a baseline level of mitotic aberrations, cells expressing BRCA1^4P^ had significantly (>3-fold) increased levels above wild-type and a trend of ~30% increased levels above vector control (Figure 5B), suggesting that BRCA1^4P^ produces more insult to mitosis than having no BRCA1 at all. As evidenced by the observed increase in mitotic aberrations (rosettes and bridges), BRCA1^4P^ cells struggle through mitosis and experience death through mitotic catastrophe. Thus, it seems as if BRCA1^4P^ causes a severe reduction in HRR concomitant with a defective G2/M checkpoint and an increase in unrepaired DSBs, providing a plausible mechanism for the increased mitotic aberrations.

### BRCA1 (4P) shifts DSB repair from HRR to NHEJ in a compensatory manner

**Figure 6:**
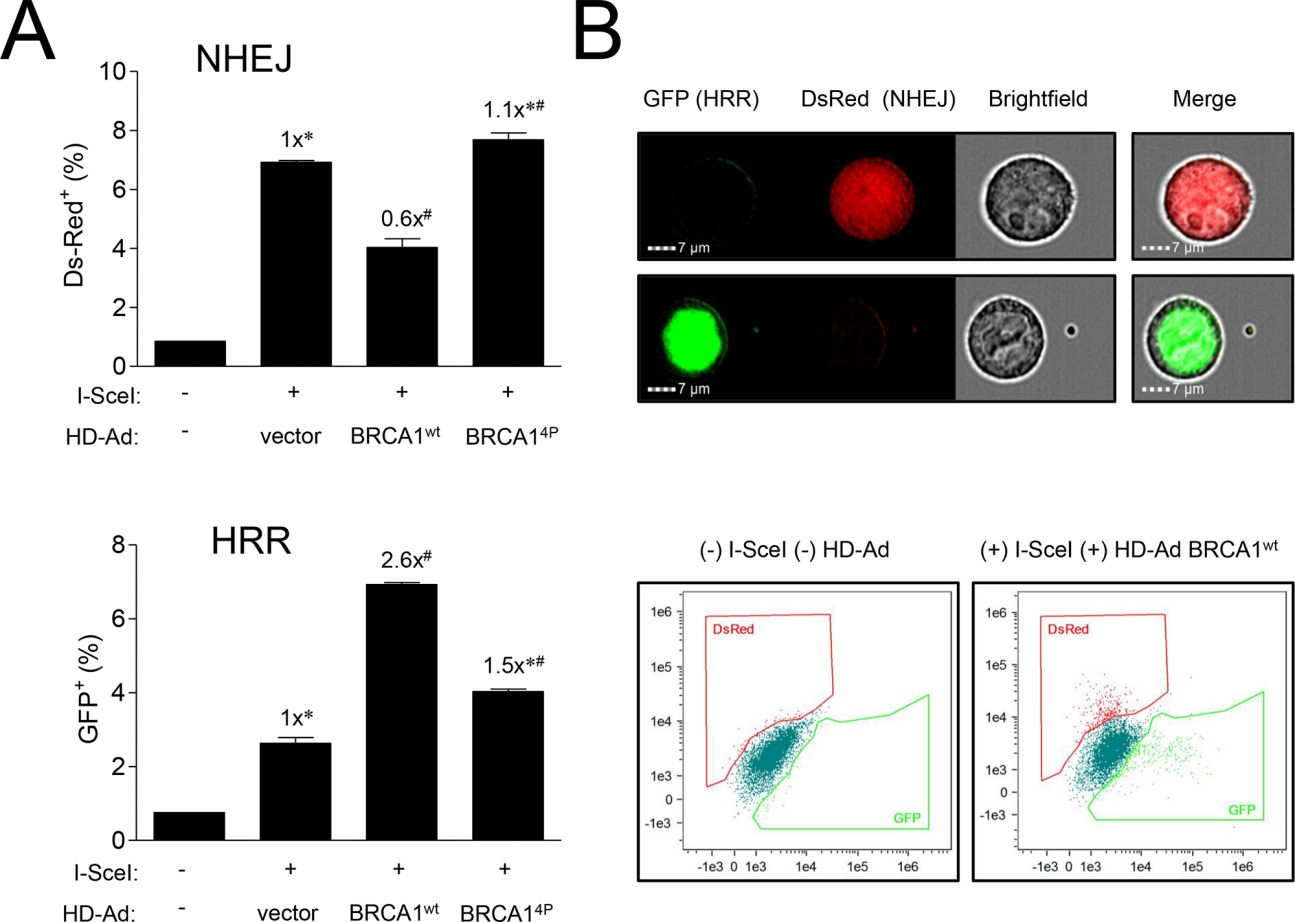
Mutations of BRCA1 phosphorylation sites inversely affect different pathways of DSB repair **A.** HCC1937-HRR/NHEJ cells were infected with HD-Ad vectors followed 48 hours later by infection with Ad-SceI as indicated. Thirty-six hours after Ad-SceI infection, 5000 cells were analyzed for GFP (HRR) and DsRed (NHEJ) fluorescent events using an imaging flow cytometry system. Error bars show the SEM from 3 independent experiments. *F*(2,6) = 77.80, *p* = < 0.0001 for DsRed and *F*(2,6) = 452.4, *p* = < 0.0001 for GFP. *p < 0.05 relative to BRCA1^wt^,^#^ *p* < 0.05 relative to vector control. **B.** Representative images of *green* GFP (HRR) and *red* DsRed (NHEJ) fluorescent cells counted in panel A. *Brightfield* images show cell shape. Representative histograms from uninfected control and infected (HD-Ad BRCA1^wt^ + Ad-SceI) cells are shown.

As DSBs are repaired by multiple pathways, we hypothesized that the HRR-deficient BRCA1^4P^ cells could be attempting to repair damage by some other means, most likely NHEJ. To explore this notion, we revisited the DR-GFP repair assay in HCC1937 cells and included a DsRed reporter which scores for NHEJ. This system allows us to simultaneously determine both HRR (GFP) and NHEJ (DsRed) events across the same cell population, providing a method to determine if NHEJ might compensate for the defective HRR observed with BRCA1^4P^. DSB repair was examined at an earlier time point in these experiments as NHEJ kinetics are generally faster than those for HRR [34]. Comparing wild-type and BRCA1^4P^ cells, we found an inverse relationship between HRR and NHEJ; as HRR decreased in the BRCA1^4P^ cells, there was a concomitant increase in NHEJ, with the inverse relationship observed in wild-type cells (Figure 6A, with representative flow cytometry images and histograms shown in Figure 6B). Thus, the lack of BRCA1 SQ-cluster phosphorylation catalyzes a shift from HRR to more error-prone NHEJ. This shift in the quality of DSB repair, coupled with an inadequate G2/M arrest, permits excessively damaged cells to inappropriately attempt mitosis, thus facilitating chromosomal instability and resulting in mitotic catastrophe.

## DISCUSSION

It was previously suggested that the radiosensitivity of BRCA1-defective cells is not entirely attributable to impaired cell cycle checkpoints [25]. Rather, some function of BRCA1 - other than its intra-S and/or G2/M checkpoint activity - affects cell survival after IR. BRCA1 is known to function and play an important role during mitosis by preventing inappropriate centrosome amplification via the interaction of hypo-phosphorylated BRCA1 with γ-tubulin [35, 36]. Furthermore, recent findings have demonstrated that ATM is activated during normal mitosis in the absence of any exogenous DNA damage suggesting a role for ATM in mitotic processing [37, 38]. Therefore, lack of BRCA1 SQ-cluster phosphorylation is likely to affect mitosis and beyond. The results presented herein demonstrate that BRCA1^4P^, with all four major SQ-cluster serine residues mutated to alanines mimicking un-phosphorylated BRCA1, impairs effective HRR and promotes aberrant mitotic progression.

While we found that the S1387A mutant alone resulted in a modest decrease in HRR, additional serine-to-alanine changes caused significantly more pronounced effects and further reduced repair to vector control levels. This finding suggests that abrogation of the intra-S checkpoint in cells expressing S1387A does not influence overall HRR levels to any major extent, despite the critical nature of this type of DSB repair during DNA replication [25]. The greatest effect on HRR was seen with S1387A in combination with S1423A, a mutation known to abrogate the G2/M checkpoint [24]. This result suggests that not permitting sufficient time in G2 for appropriate repair presents a considerable impediment to maintaining chromosomal integrity prior to mitosis. Additional alterations to alanine at S1457 and S1524, predominantly phosphorylated by ATR [20, 23], did not further reduce HRR levels. However, we cannot rule out individual roles of either one of these two sites in HRR since they were only included in the present study as part of BRCA1^4P^.

BRCA1, together with CHK1, are believed to control exit from mitosis, and the inhibition of either can cause mitotic catastrophe [39]. It was demonstrated that when either protein was reduced by siRNA silencing, cells continued to cycle without dividing, forming multinucleated cells. The fate of such multinucleated cells is known to be under p53 control [40]. Interestingly, a previous report found that a triple SQ-cluster BRCA1 mutant (S1387/1423/1524A) did not affect BRCA1 foci formation but did result in a robust G1/S checkpoint arrest in response to IR [41]. In light of our findings, this could be explained by proposing that damaged cells expressing the triple mutant undergo aberrant mitosis but arrest in G1/S due to wild-type p53 in the MCF-7 cells used in that study. Additional work using p53-defective cells with an abrogated G1/S checkpoint (such as the HCC1937 and UWB1.289 cells used here) has shown that these undivided, damaged 4N cells will enter S-phase again, replicating to 8N and beyond until the cells arrest or die [42]. It has also been suggested that mutant p53 tumor cells lack a mitotic checkpoint [43]. Thus, the effect of mutating critical phosphorylation sites within BRCA1, particularly at S1387 and S1423, results in the abrogation of the intra-S and G2/M checkpoints, causing erroneous mitotic entry and exit which results in the generation of aneuploid, undivided “daughter” cells. We found in the present study that such cells with mitotic aberrations (bridges and rosettes) appear in BRCA1^4P^ cells to a greater extent than wild-type cells even in the absence of any DNA damage. These BRCA1^4P^ cells would eventually die by mitotic catastrophe.

Notably, we observed more GFP^+^ events in cyclin A^+^ cells (HRR^+^ in late S/G2) with BRCA1^4P^ compared to wild-type, even though the overall level of HRR was lower in the BRCA1^4P^ cells (see Figure 4). As such, our results could be explained by the idea that as BRCA1^4P^ cells are defective in all checkpoints, the corresponding GFP^+^ cells either fail to progress through mitosis and die or are eliminated via other mechanisms once they have begun a new cell cycle. Alternatively but not mutually exclusive, the absolute reduction in HRR^+^ cells could also be caused by the re-direction of DSB repair from HRR to NHEJ at the I-*Sce*I-cut DR-GFP cassette, thus resulting in fewer GFP^+^ cells. This scenario is supported by the simultaneous increase in DsRed^+^ cells seen with BRCA1^4P^ from a separate I-*Sce*I repair cassette not affected by competing HRR at the same DSB (in DR-GFP).

As BRCA1 is directed to sites of DSBs where it recruits and is phosphorylated by ATM [44], differential BRCA1 phosphorylation may therefore be the critical upstream event which sets the stage for subsequent steps in the DDR such as cell cycle arrest and DSB repair pathway choice [6, 21]. More specifically, SQ-cluster phosphorylation might indirectly influence alternative protein binding to BRCA1 via BRCT through cell cycle-dependent phosphorylation of BACH1 and CtIP via CDKs which occurs during the S and G2 phases of the cell cycle, respectively, and is a prerequisite for the BRCT interaction [14, 16, 45–47]. Additional studies will be needed to determine if specific phosphorylation patterns trigger the initial events involved in the DSB repair activity of BRCA1.

Previous work has shown that the BRCA1 RING domain-associated ubiquitin ligase activity acts upstream of the BRCT domain-mediated HRR activity [48], although more recent studies suggest that BRCA1/BARD1-directed ubiquitination is not essential *in vivo* for either HRR [49] or the suppression of tumorigenesis [50]. Nevertheless, a hierarchal model can be proposed whereby BRCA1 phosphorylation leads to the activation of BRCA1 ubiquitin ligase activity important for guiding BRCT-mediated repair processes. Interestingly, whereas SQ-cluster mutations cause HRR failure, specific mutations in the BRCT domain result in aberrant hyper-recombination [28]. A possible explanation for this observation is the inability of the RAP80 A complex to bind BRCA1 and limit DNA end resection [51, 52], not by CtIP-MRN (also unable to bind BRCA1) but through the Exo1/Dna2 nucleases, resulting in excessive ssDNA at the DSB and subsequent aberrant hyper-recombination [28]. A similar phenotype was seen when RAP80 or Abraxas were silenced [51, 52]. This hierarchal model is further supported by the finding that mutating the BRCA1 RING domain restored normal levels of HRR to a BRCT mutant causing aberrant hyper-recombination [28]. Thus, BRCA1 phosphorylation might regulate ubiquitin ligase activity which in turn enforces the quality of DSB repair. The interaction of BRCA1 with PALB2 seems to occur normally when BRCA1 is in an un-phosphorylated state as BRCA1^4P^ binds PALB2 indistinguishably from wild-type BRCA1 in our hands in agreement with a previous study that determined the effect of single S→ A alterations at either S1387, S1423, or S1524 on PALB2 binding [18]. Additional studies will determine if phospho-mimetic S→D BRCA1 mutations are able to bind PALB2 or not.

Altogether, our findings suggest that multiple mutations within the BRCA1 SQ-cluster domain results in mitotic failure, even in unchallenged cells. This failure is the result of a collapse of two intertwined processes: an inability to conduct high-fidelity DSB repair via HRR and an ineffective G2/M checkpoint allowing damaged cells to go to—and through—mitosis. Combined, these breakdowns result in mitotic aberrations and catastrophic death. In short, cells perceive un-phosphorylated BRCA1 (or S→ A surrogate alterations) as a “green light” for cell cycle progression regardless of chromosomal integrity status.

## MATERIALS AND METHODS

### Cell culture

HCC1937 cells [29] were cultured at 37°C in RPMI 1640 (Life Technologies, Grand Island, NY, USA) supplemented with 10% FBS and antibiotics. UWB1.289 BRCA1-null and BRCA1 wild-type complemented ovarian cancer cells [32] (American Type Culture Collection, Manassas, VA, USA) were grown at 37°C in 50% RPMI 1640 + 50% MEGM (Lonza Inc., Allendale, NJ, USA), supplemented with 3% FBS and antibiotics. The HCC1937 cells have not been authenticated whereas the UWB1.289 cells and derivative were authenticated by the ATCC.

### Plasmids

pcDNA3(*Bss*HII)-HA-3xFLAG-BRCA1 S1387A (BRCA1^1P^) and S1387/1423A (BRCA1^2P^) were generated from pcDNA3-HA-BRCA1 plasmids harboring the indicated serine-to-alanine alterations [24, 25, 53] by swapping the *Bam*HI-*Xho*I fragments with pcDNA3(*Bss*HII)-HA-3xFLAG-BRCA1 wild-type [28]. The S1387/1423/1457/1524A quadruple mutant (BRCA1^4P^) was then generated from plasmid pcDNA3(*Bss*HII)-HA-3xFLAG-BRCA1 S1387/1423A (BRCA1^2P^) by sequential rounds of QuikChange site-directed mutagenesis (Stratagene—Agilent Technologies, Santa Clara, CA, USA) using primers 5′–GCAGTATTAACTGCACAGAAAAGTAGTG–3′ and 5′–CACTACTTTTCTGTGCAGTTAATACTGC-3′ to create the S1457A mutation and primers 5′-GAATAGAAACTACCCAGCTCAAGAGGAGCTC-3′ and 5′-GAGCTCCTCTTGAGCTGGGTAGTTTCTATTC-3′ to create the S1524A mutation. Mutations were confirmed by DNA sequencing. The Myc (5x)-PALB2 plasmid was generated by cloning the 5x-Myc tag from Myc-CtIP [28] into pCMV-SPORT6-PALB2 (GeneCopoeia, Rockville, MD, USA).

### Generation of viral vectors and stably infected cell lines

Helper-dependent adenovirus (HD-Ad) vectors were generated as described [28]. Briefly, pcDNA3(*Bss*HII)-HA-3xFLAG-BRCA1 plasmids were digested with *Bss*HII and the HA-3xFLAG-BRCA1 fragments ligated into the *Asc*I site of the HD-Ad plasmid pΔ28E4LacZ. The resulting pΔ28E4LacZ-HA-3xFLAG-BRCA1 plasmids were digested with *Pme*I to release the viral DNA from the plasmid backbone, and viral DNA was packaged into adenovirus and amplified by transfecting HEK293–116C cells using Superfect (Qiagen, Valencia, CA, USA) as described. Stably infected UWB1.289-Fucci cells were created by PCR of the Fucci cassette from pFucci-S/G2/M-Green (Amalgam, MBL International Corp., Woburn, MA, USA) using *Bam*HI (5′) and *Eco*RI (3′) modified primers followed by cloning into *Bam*HI-*Eco*RI cut pWPXLd (Addgene plasmid #12258, Cambridge, MA, USA). Lentivirus was generated by co-transfection with pMD2.G (Addgene plasmid #12259) and psPAX2 (Addgene plasmid #12260) in HEK293T cells as described [54].

### DNA repair assays

The HRR-GFP and NHEJ-DsRed repair systems have been described previously [28, 54]. Separate I-*Sce*I repair cassettes score for HRR and NHEJ independently. Briefly, HCC1937/DR-GFP cells were transduced with a NHEJ-DsRed lentivirus to generate HCC1937-HRR/NHEJ [28]. HCC1937-HRR/NHEJ cells at ~70–90% confluencey were infected with BRCA1 HD-Ad vectors. Forty-eight hours after HD-Ad infection, cells were infected with an adenovirus expressing the endonuclease I-*Sce*I (Ad-SceI) and repair events determined at 36 or 72 hours after Ad-SceI infection by either the Amnis ImageStream (Seattle, WA, USA) microscopy/flow system or the BD FACSAria (San Jose, CA, USA) fluorescence-activated cell sorting (FACS) flow cytometer.

### Mitotic aberration assay

UWB1.289 cells were grown in 12-well tissue culture plates and infected with the HD-Ad vectors. Cells were fixed in 100% methanol 72 hours post-infection. Giemsa solution (Sigma-Aldrich, St. Louis, MO, USA) was diluted 1:20 in de-ionized water and cells were stained for ~60 minutes, after which time they were washed, dried, and imaged using a bright-field microscope. Images were manually analyzed and mitotic aberrations were recorded.

### Cell viability assay

UWB1.289 (parental) or BRCA1-complemented (stable) cells were serially diluted and treated with mitomycin C. For the parental HD-Ad-infected set, transduction was performed 24 hours prior to re-seeding in 96-well tissue culture plates. Toxicity was determined using the CellTiter-Glo^®^ Luminescent Cell Viability Assay kit (Promega, Madison, WI, USA) following the manufacturer's protocol.

### Live-cell imaging

Live-cell imaging was performed as described [55], using a Zeiss (Jena, Germany) Cell Observer SD spinning disk confocal microscope. In brief, UWB1.289 cells were stably transduced with a lentivirus construct (WPXLd-H2B-mRFP-12A-puro) expressing histone H2B-mCherry derived from pWPXLd, L087 RRI-Red (GeneBank: EU424173.1), and pcDNA3-H2B-mCherry (Addgene plasmid #20972) to visualize chromatin dynamics. Cells were transferred to coated glass-bottom optical dishes and infected with HD-Ad vectors 48 hours prior to recording. Cells were kept on an incubated stage at 37°C and 5% CO_2_ for the entirety of the experiment. Videos were analyzed using PerkinElmer's Volocity software (Waltham, MA, USA).

### Antibodies

Antibodies used were FLAG (M2; Sigma-Aldrich), Myc (9B11; Cell Signaling Technology, Danvers, MA, USA), RAD51 (Ab-1; Calbiochem—Merck Millipore, Billerica, MA, USA), DNA-PKcs (4F10C5; BD Pharmigen, San Diego, CA, USA), α-tubulin (3873S; Cell Signaling Technology), and Cyclin A (H-432; Santa Cruz Biotechnology, Dallas, TX, USA).

### Immunocytochemistry

UWB1.289 cells were seeded onto 4-well chamber slides and infected with HD-Ad vectors. Forty-eight hours after infection the cells were irradiated with 4 Gy using a Gammacell 40 Exactor (Nordion, Ottawa, ON, Canada) or left un-irradiated, fixed with 3% paraformaldehyde after 8 hours of incubation, permeablized with 0.5% Triton X-100, immuno-labeled, and counterstained with DAPI to reveal cell nuclei. For RAD51 foci analysis, cells were imaged at 63x magnification using a Zeiss LSM 710 META confocal laser scanning microscope running ZEN 2011 software. Images were quantified with Volocity software using packaged analysis protocols to objectively measure the number of RAD51 foci per DAPI-stained nucleus. Manual counting and quantification were used for all other confocal image analyses.

### Western blotting

Western blotting was performed as previously described [28], with additional modifications. Cells were lysed in RIPA buffer supplemented with HALT™ protease and phosphatase inhibitors (Sigma-Aldrich). Proteins were separated on Criterion™ TGX gels (Bio-Rad Laboratories, Hercules, CA, USA) and transferred to PVDF membranes, which were exposed to primary antibodies at a 1:1000 dilution. Protein bands were detected using infrared-emitting conjugated secondary antibodies, either anti-rabbit DyeLight 800 (Rockland Immunochemicals, Gilbertsville, PA, USA) or anti-mouse Alexa 680 (Life Technologies, Grand Island, NY, USA) with the Odyssey infrared imaging system from Li-Cor Biosciences (Lincoln, NE, USA).

### Immunoprecipitation

Recombinant HA-3xFLAG-BRCA1 (wild type or phospho-mutant) and Myc-PALB2 were immunoprecipitated as described previously [28].

### Statistics

Student's unpaired, two-tailed *t*-tests for paired data and one-way ANOVAs followed by Student Neuman-Keuls post-hoc test for multiple comparisons were performed on data sets using GraphPad Prism 5.0 (GraphPad Software, Inc., La Jolla, CA, USA). A value of *p* < 0.05 was considered significant.

## SUPPLEMENTARY FIGURES AND VIDEO


