# Correction: Mutation of the BRCA1 SQ-cluster results in aberrant mitosis, reduced homologous recombination, and a compensatory increase in non-homologous end joining

**DOI:** 10.18632/oncotarget.11846

**Published:** 2016-09-05

**Authors:** Jason M. Beckta, Seth M. Dever, Nisha Gnawali, Ashraf Khalil, Amrita Sule, Sarah E. Golding, Elizabeth Rosenberg, Aarthi Narayanan, Kylene Kehn-Hall, Bo Xu, Lawrence F. Povirk, Kristoffer Valerie

Present: Due to an error made during manuscript preparation, one of Dr. Ashraf Khalil's affiliations was omitted from the final version of the article.

Corrected: The correct affiliation list for this manuscript is provided below. Publisher sincerely apologizes for this oversight.

Original article: Oncotarget. 2015; 6(29): 27674-87. doi: 10.18632/oncotarget.4876.

Jason M. Beckta^1,2,*^, Seth M. Dever^1,2,*^, Nisha Gnawali^1^, Ashraf Khalil^1,7^, Amrita Sule^1,2^, Sarah E. Golding^1^, Elizabeth Rosenberg^1^, Aarthi Narayanan^3^, Kylene KehnHall^3^, Bo Xu^4^, Lawrence F. Povirk^5^, Kristoffer Valerie^1,2,6^

^1^ Department of Radiation Oncology, Virginia Commonwealth University, Richmond, VA, USA

^2^ Department of Biochemistry and Molecular Biology, Virginia Commonwealth University, Richmond, VA, USA

^3^ National Center for Biodefense and Infectious Diseases, George Mason University, Manassas, VA, USA

^4^ Cancer Research Department, Southern Research Institute, Birmingham, AL, USA

^5^ Department of Pharmacology and Toxicology, Virginia Commonwealth University, Richmond, VA, USA

^6^ Massey Cancer Center, Virginia Commonwealth University, Richmond, VA, USA

^7^ Department of Biochemistry, National Liver Institute, Menoufiya University, Egypt

^*^ These authors have contributed equally to this work

