# Supplementary material for: Mutation of the BRCA1 SQ-cluster results in aberrant mitosis, reduced homologous recombination, and a compensatory increase in non-homologous end joining
Source: Oncotarget. 2015 Aug 12;6(29):27674–87. doi: 10.18632/oncotarget.4876 (PMC4695017; doi:10.18632/oncotarget.4876)
Supplement: Supplementary file 1 [file oncotarget-06-27674-s001.pdf]

SUPPLEMENTARY FIGURES AND VIDEO

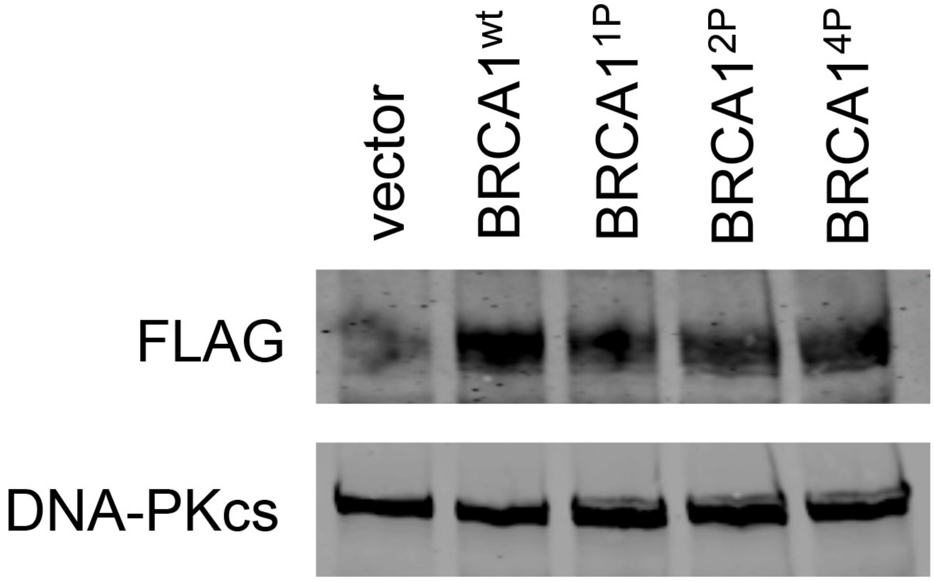

Supplementary Figure S1: FLAG-tagged BRCA1 is efficiently and equally expressed from HD-Ad vectors in infected HCC1937 cells. HCC1937 cells were harvested 48 hours after infection with the indicated HD-Ad vectors to examine FLAG-BRCA1 expression by western blot analysis. DNA-PKcs was used as a loading control.

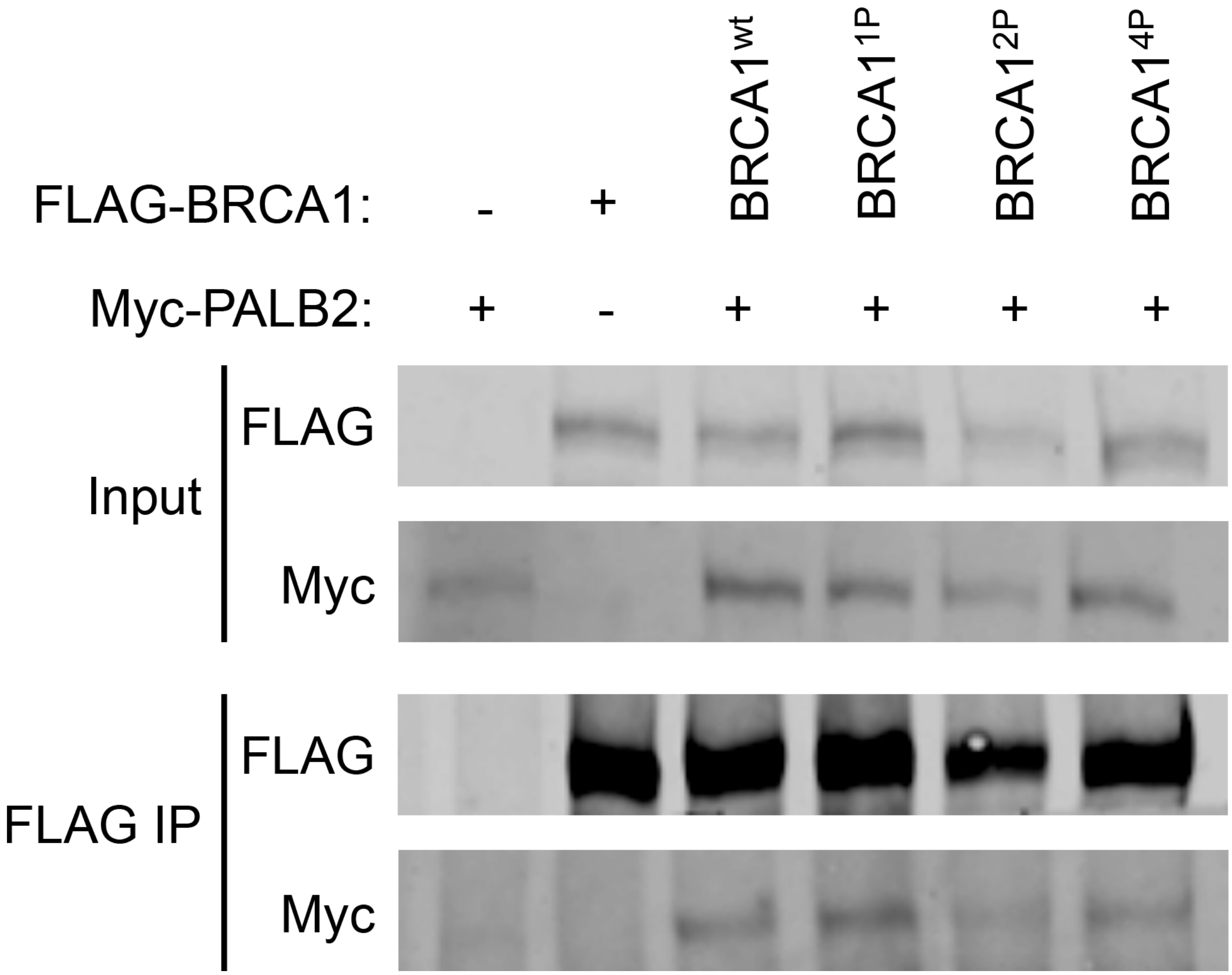

Supplementary Figure S2: PALB2 binding to BRCA1 is not dependent on BRCA1 SQ-cluster phosphorylation. HEK293T cells were co-transfected with the indicated FLAG-BRCA1 and Myc-PALB2 plasmid constructs and harvested 48 hours after transfection. Lysates were subject to FLAG immunoprecipitation followed by western blot analysis with the indicated antibodies.

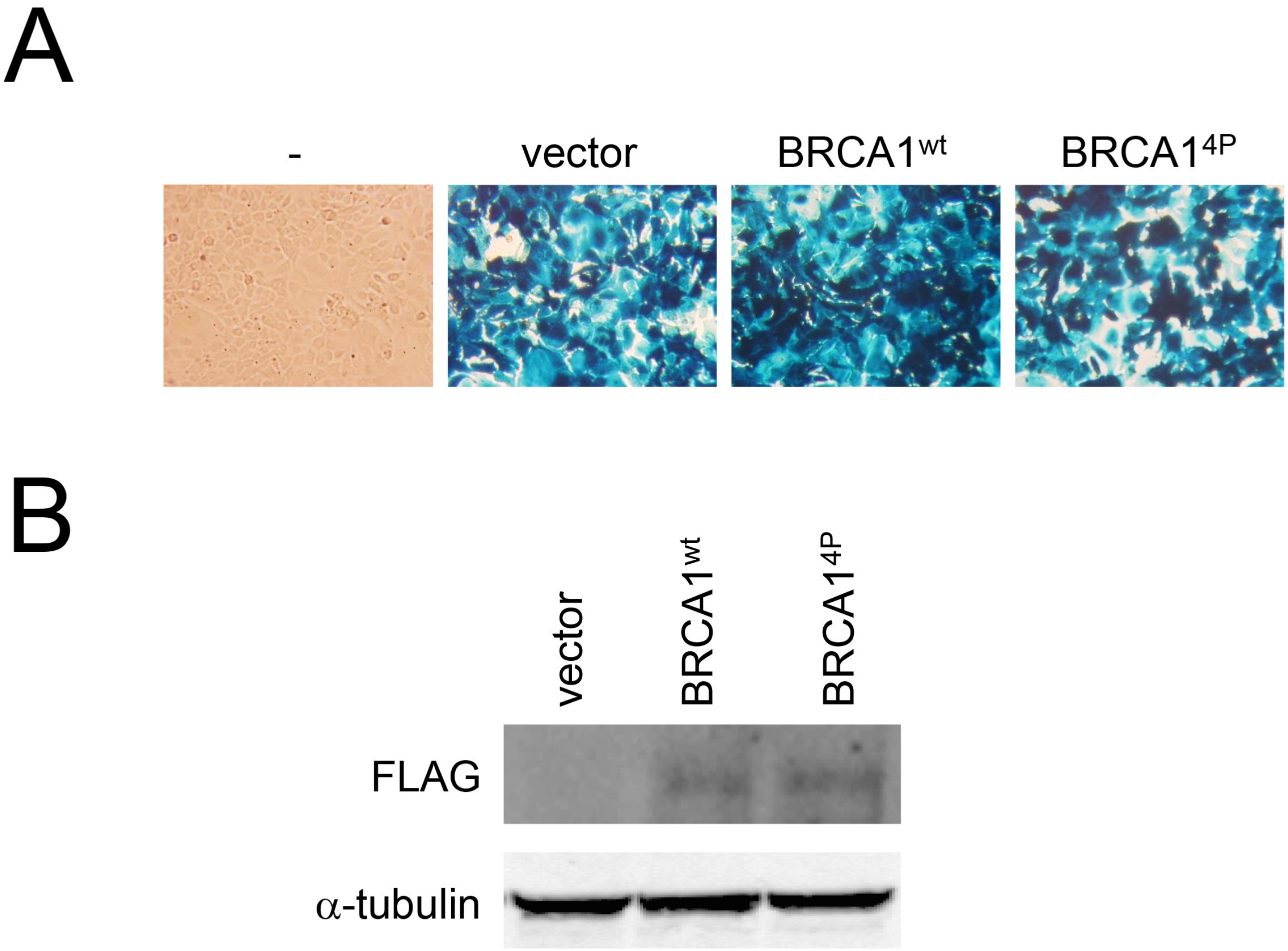

Supplementary Figure S3: HD-Ad vectors efficiently infect and express FLAG-tagged BRCA1 in UWB1.289 cells. A. UWB1.289 cells were infected with the indicated HD-Ad vectors and stained for β-galactosidase reporter activity 48 hours after infection. Representative *brightfield* images are shown. **B.** FLAG-BRCA1 expression from HD-Ad vectors in UWB1.289 cells by western blot analysis. α-Tubulin was used as a loading control.

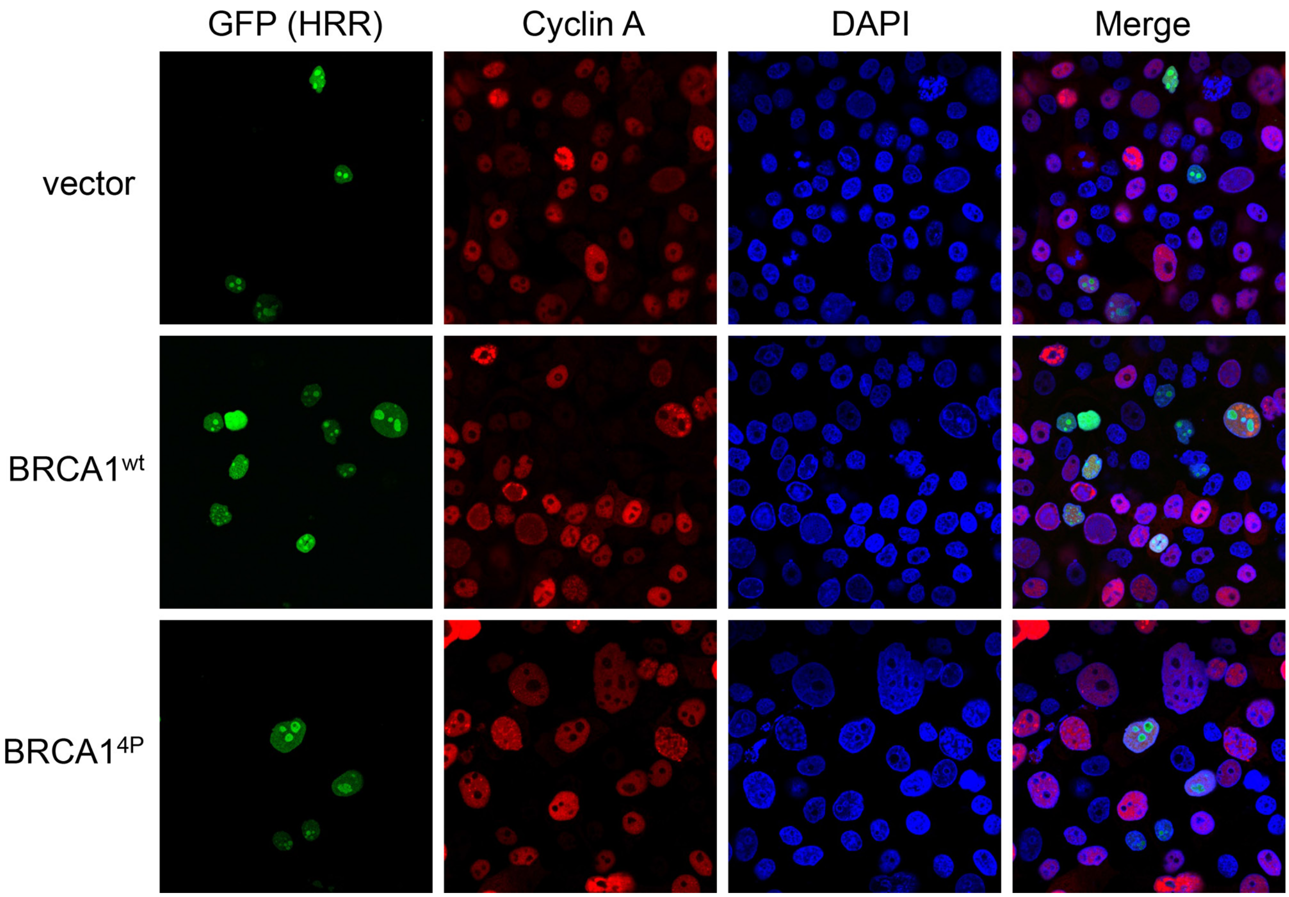

Supplementary Figure S4: Correlation of GFP (HRR) and cyclin A expression in HCC1937/DR-GFP cells. Representative images of cells from Figure 4B showing GFP (*green*) fluorescence and immunolabeled cyclin A (*red*) co-expression. DAPI (*blue*) staining indicates cell nuclei.

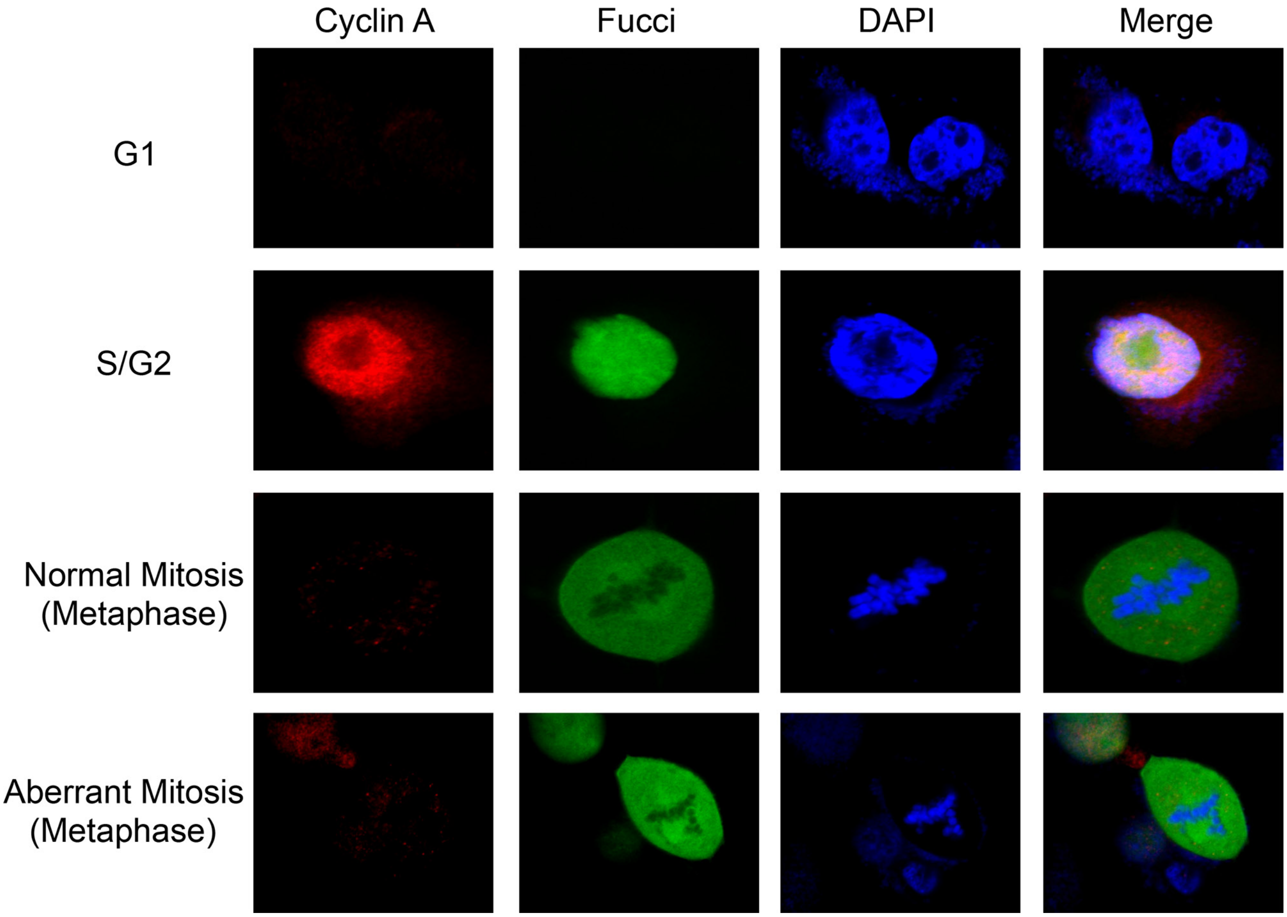

Supplementary Figure S5: Fucci-positive cells which are not cyclin A-positive are in mitosis. UWB1.289/Fucci cells showing Fucci (*green*) fluorescence and immunolabeled cyclin A (*red*) co-expression at the indicated cell cycle phases. DAPI (*blue*) staining indicates cell nuclei.

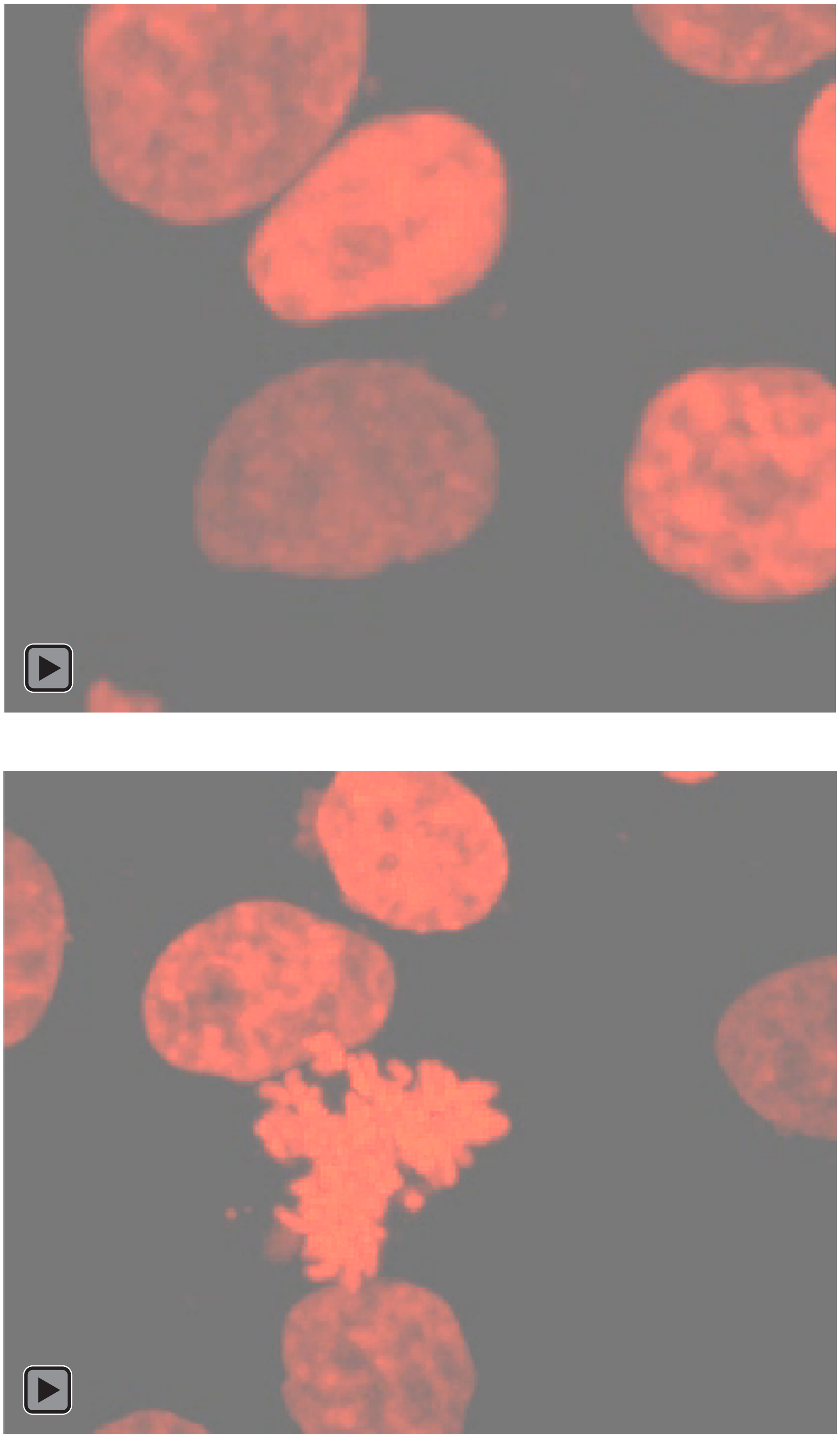

Supplementary Figure S6: Live-cell video recordings of parental UWB1.289/H2B-mCherry cells undergoing normal and prolonged, aberrant mitosis.
